# Neurogranin regulates CaM dynamics at dendritic spines

**DOI:** 10.1038/srep11135

**Published:** 2015-06-18

**Authors:** Amber Petersen, Nashaat Z. Gerges

**Affiliations:** 1 Department of Cell Biology, Neurobiology and Anatomy, the Medical College of Wisconsin, Milwaukee, WI 53132.

## Abstract

Calmodulin (CaM) plays a key role in synaptic function and plasticity due to its ability to mediate Ca^2+^ signaling. Therefore, it is essential to understand the dynamics of CaM at dendritic spines. In this study we have explored CaM dynamics using live-cell confocal microscopy and fluorescence recovery after photobleaching (FRAP) to study CaM diffusion. We find that only a small fraction of CaM in dendritic spines is immobile. Furthermore, the diffusion rate of CaM was regulated by neurogranin (Ng), a CaM-binding protein enriched at dendritic spines. Interestingly, Ng did not influence the immobile fraction of CaM at recovery plateau. We have previously shown that Ng enhances synaptic strength in a CaM-dependent manner. Taken together, these data indicate that Ng-mediated enhancement of synaptic strength is due to its ability to target, rather than sequester, CaM within dendritic spines.

Calmodulin (CaM) is one of the most important regulatory proteins that mediates responses to Ca^2+^ flux and modulates the activity of many signaling molecules in the cell. At dendritic spines, there are multiple CaM targets that are crucial for synaptic plasticity, a widely accepted cellular correlate of learning and memory formation^1^,^2^,^3^. Some of these targets even have opposing functions. For example, with a relatively large increase in local Ca^2+^ over a short period of time (a few seconds), Ca^2+^ binding causes a conformational change in CaM that allows it to activate Ca^2+^/CaM-dependent protein kinase II (CaMKII), which mediates α-amino-3-hydroxy-5-methyl-4-isoxazolepropionic receptor (AMPAR) delivery to synapses and the expression of long-term potentiation (LTP)^4^,^5^,^6^,^7^. On the other hand, a small increase in postsynaptic Ca^2+^ causes CaM to activate calcineurin, resulting in the expression of long-term depression (LTD)^8^,^9^,^10^. It is well appreciated that the total concentration of CaM-binding proteins is much higher than that of CaM itself, indicating that CaM is a limiting factor in the cell^11^,^12^. Thus, the regulation of its availability is essential for the activation of CaM-dependent signaling pathways. Some models assume that under resting conditions CaM is freely diffusible^13^ whereas other studies in smooth muscle cells and fibroblasts suggest that CaM is largely immobile^14^,^15^,^16^,^17^ Importantly, CaM mobility and diffusion in dendritic spines has not been addressed.

Under resting conditions, Ca^+2^-free CaM (apo-CaM) binds to a group of proteins called calpacitins. For example, GAP43 is a calpacitin which exists only presynaptically, and neurogranin and pep19 are calpacitins which exist solely postsynaptically. Two main views exist regarding the relevance of such binding. According to one view, calpacitins sequester CaM and thereby inhibit their ability to activate subsequent targets^18^,^19^. The other view is that calpacitins may target and/or concentrate CaM to facilitate Ca^2+^/CaM-mediated signaling at specialized localizations^20^,^21^,^22^. In support of the latter view, our lab has shown that overexpression of Neurogranin (Ng), a postsynaptic calpacitin that is abundant in CA1 pyramidal neurons^23^,^24^,^25^, increases CaMKII activation and enhances synaptic strength^26^. Importantly, Ng mutants that are incapable of binding to CaM and those that constitutively bind to CaM even at high Ca^2+^ levels, are incapable of enhancing synaptic strength^26^. Additionally, we have shown that increasing Ng shifts the CaM localization close to the plasma membrane within dendritic spines^27^. Collectively, these data suggest that Ng may regulate CaM diffusion within dendritic spines. Despite the plethora of studies on CaM signaling in neurons, questions concerning CaM diffusion at dendritic spines and whether Ng regulates such diffusion remain unanswered.

Fluorescence recovery after photobleaching (FRAP) has been used to gain valuable insight into the diffusibility of several proteins at dendritic spines in intact living neurons^28^,^29^,^30^. This approach involves tagging the protein of interest with a fluorophore and irreversibly photobleaching a portion of the fluorophores in a region of interest, in this case, the dendritic spine. The recovery of fluorescence in the bleached region reflects the mobility of the fluorophores. Thus, FRAP analysis can provide valuable information about the diffusion characteristics of molecules (e.g. diffusion rate and the plateau value, or final percent recovery, which is a measure of the fraction of molecules that are mobile on the timescale of the measurement).

In this study, we have used live cell confocal microscopy to explore the dynamics of CaM in dendritic spines using FRAP. Our results show that only a small fraction of CaM is immobile in spines. We have also investigated the role of Ng and its phosphorylation state in regulating CaM dynamics. We show that Ng regulates CaM diffusion rate, but not its immobile fraction.

## Materials and Methods

### Animals and Hippocampal Slice Preparation

Sprague-Dawley rats purchased from Charles River Laboratories (Portage, MI, USA) were maintained on a 12 h light/dark cycle (lights off at 6:00 pm). Organotypic hippocampal slices were prepared as previously described^31^ at postnatal day 5 or 6. All biosafety procedures and animal care protocols described here were approved by the Medical College of Wisconsin Institutional Animal Care and Use Committee and were performed in strict accordance with the Guidelines for Care and Use of Laboratory Animals of the National Institutes of Health.

### DNA Constructs and Expression

Calmodulin, neurogranin and postsynaptic density protein-95 were cloned from commercial rat brain cDNA (Clonetec). In-frame EGFP fusion proteins were made in the EGFP-C1 plasmid. The EGFP sequence was removed by restriction digest and replaced with CFP or YFP sequences. All recombinant plasmids have been verified with DNA sequencing. Constructs were expressed in organotypic hippocampal slices after 4–8 days in culture via the biolistics transfection system^32^.

### Confocal Imaging

All imaging experiments were done with live organotypic hippocampal slices in artificial cerebral spinal fluid (ACSF) (119 mM NaCl, 2.5 mM KCl, 4 mM CaCl_2_, 4 mM MgCl_2_, 26 mM NaHCO_3_, 1 mM NaH_2_PO_3_, 1 mM glucose) gassed with 5% CO_2_, 95% O_2_ and maintained at 37 °C in a live imaging chamber. A Leica TCS SP5 confocal microscope was used to acquire all images and perform all bleaching experiments. All images were acquired with a 63x oil immersion lenses. The fluorescence recovery after photobleaching (FRAP) wizard provided with the Leica software was used for all FRAP experiments. The selected bleached region was centered on a spine. Bleaching was performed with the relevant laser set to 100% and zoomed-in to focus on the region of interest for 40 scans at 0.15 s intervals. Baseline fluorescence was measured from five images acquired prior to bleaching and fluorescence recovery was measured at various time intervals after bleaching. Fluorescence intensity was measured using ImageJ software (Bethesda, MD). Background fluorescence values were subtracted from measurements of both the spine and a nearby unbleached dendritic region at each time point. Recovery curves from average fluorescence at each time point were analyzed using GraphPad Prism (San Diego, CA).

Spine-to-dendrite ratios were measured as previously described^26^,^33^. Briefly, Z-stack images of dendrites were acquired with 0.2 μm steps and a maximum projection was created with Image J. Fluorescence intensity across a section of dendrite, spine and adjacent background was quantified. Average background fluorescence of each image was subtracted from both spine and adjacent dendrite averages before spine values were normalized to dendrites.

The width of dendritic spine heads and shafts and total spine length were measured from z-stack images obtained as previously described. Leica Application Suite X software was used to measure spine dimensions. All spines on an imaged section of dendrite were imaged to obtain a single value per dimension per dendrite. These values were then averaged to find the average spine head and shaft width and total spine length for each condition.

### Statistical Analysis

For FRAP experiments, non-linear regression with exponential one-phase decay was used to find a best-fit line. Unpaired t-tests were performed to compare immobile fractions, recovery constants (K), and the average recovery value at 1.8–2.8 s between conditions. The normalized spine-to-dendrite ratios for each condition were averaged and compared using the unpaired t-test. Dimensions of dendritic spine heads and shafts were compared using the Kruskal-Wallis One Way ANOVA on Ranks.

## Results

### Only a small fraction of calmodulin is immobile

**Figure 1:**
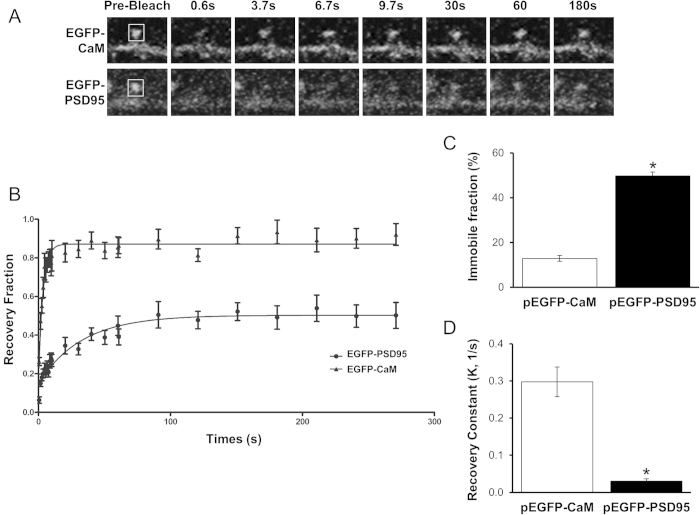
A Small Fraction of CaM in Dendritic Spines is Immobile. (**A**) Examples of dendritic spines from neurons expressing either EGFP-CaM (top) or EGFP-PSD95 (bottom) and undergoing FRAP. Representative confocal images are shown before photobleaching (“pre-bleach”), right after photobleaching (“0.6 s”) and at different times during fluorescence recovery, as indicated. Bleached regions are indicated with squares in the “pre-bleach” panels. (**B**) Recovery curves of EGFP-CaM and EGFP-PSD95 were fit by one-phase exponential equations (n = 11 EGFP-CaM, n = 21 EGFP-PSD95). (**C**) The bar graph represents the immobile fraction of EGFP-CaM and PSD95 at the plateau. The immobile fraction of EGFP-CaM is significantly smaller than that of EGFP-PSD95 (p < 0.0001). **D**) The bar graph shows the recovery constant (K) of EGFP-PSD95 (0.03 ± 0.01/s) and EGFP-CaM (0.30 ± 0.04/s, p < 0.0001).

Several models of CaM signaling predict that CaM is largely immobile in cells^14^. This is based on findings that CaM levels are lower than the levels of its binding partners, suggesting that CaM availability is limiting^11^,^12^. However, investigations into CaM diffusion have mostly been performed using non-neuronal cell cultures such as HEK293 or fibroblasts. Up to this point, the mobility of CaM in dendritic spines has not been directly assessed. To test the diffusion of CaM in dendritic spines, we have performed FRAP analysis on EGFP-CaM at the dendritic spines of CA1 hippocampal neurons. The FRAP recovery curve was fit with a one-phase exponential decay equation (Fig. 1B) to determine recovery rate and recovery plateau, which is indicative of the immobile fraction. Surprisingly, we found that only a small fraction of CaM is immobile (Fig. 1C). This is in contrast to post-synaptic density protein-95 (PSD95), a scaffolding protein that is known to have a significant immobile fraction at dendritic spines. EGFP-PSD95 has a significantly larger immobile fraction (p < 0.0001) than that of EGFP-CaM (Fig. 1C). The rate constant of the recovery (K) was also faster for CaM relative to PDS95 (0.2976 ± 0.0399/s and 0.0303 ± 0.0061/s, respectively, p < 0.0001, Fig. 1D). These data suggest that only a small fraction of CaM is immobile within spines.

### Neurogranin slows calmodulin diffusion rate but does not alter the immobile fraction

**Figure 2:**
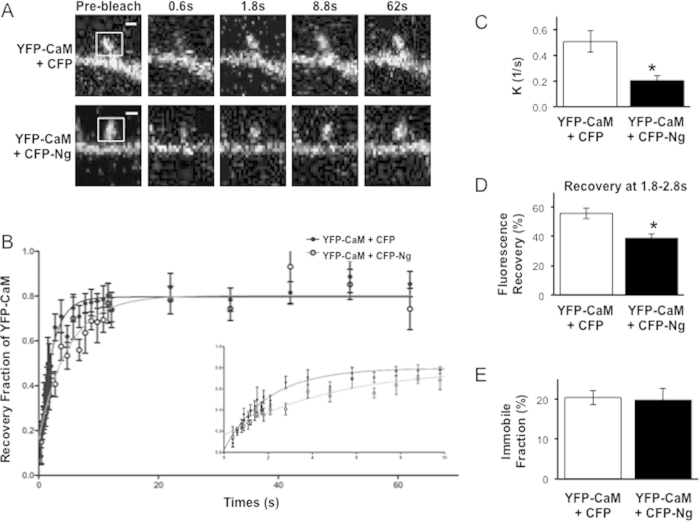
Ng Slows CaM Diffusion. (**A**) Examples of dendritic spines from neurons co-expressing YFP-CaM with either CFP alone (top) or CFP-Ng (bottom) and undergoing FRAP. Representative confocal images are shown before photobleaching (“pre-bleach”), right after photobleaching (“0.6 s”) and at different times during fluorescence recovery, as indicated. Bleached regions are indicated with squares in the “pre-bleach” panels. (**B**) Recovery curves of YFP-CaM in the presence of CFP alone or CFP-Ng were fit with a one-phase exponential decay equation (n = 10 CFP, n = 7 CFP-Ng). Inset shows the initial 10 seconds of fluorescence recovery. (**C**) The bar graph represents the recovery constant (K) of YFP-CaM in presence of CFP alone or CFP-Ng (0.51 ± 0.08/s and 0.20 ± 0.04, respectively, p < 0.05). (**D**) The bar graph shows percent recovery of YFP-CaM fluorescence at 1.8–2.8 s after photobleaching in the presence of CFP alone (55.74 ± 3.56%) or CFP-Ng (38.86 ± 3.12%, p < 0.05). (**E**) The bar graph shows the immobile fraction of YFP-CaM fluorescence at the recovery plateau in the presence of CFP alone (20.37 ± 1.78%) or CFP-Ng (19.74 ± 2.94%, p = 0.8). To note, the immobile fraction of YFP-CaM is not affected by the presence of CFP-Ng.

In CA1 pyramidal neurons, neurogranin is the most abundant postsynaptic protein that binds apo-CaM^22^,^34^,^35^,^36^,^37^. Ng binds CaM only in the absence of Ca^2+^, whereas an increase in intracellular Ca^2+^ (e.g. as a result of neuronal activity) releases CaM from Ng^23^,^24^,^38^. We have previously shown that Ng expression in CA1 pyramidal neurons enhances synaptic transmission in a manner dependent on its regulated interaction with CaM^26^. Moreover, Ng is capable of shifting a pool of CaM closer to the plasma membrane^27^. Taken together, these findings suggest that Ng binding may influence the diffusion of CaM within dendritic spines. To directly test the effect of Ng on CaM diffusion, we co-expressed YFP-CaM with either CFP-Ng or CFP alone. We then performed FRAP analysis of YFP-CaM similar to that described above. We find that the rate of YFP-CaM recovery is significantly slowed by the presence of CFP-Ng relative to that of YFP-CaM when co-expressed with CFP alone (rate constant (K) of YFP-CaM is 0.20 ± 0.03/s in presence of CFP-Ng vs. 0.51 ± 0.08/s in presence of CFP alone, p < 0.05 (Fig. 2C). Figure 2D shows that YFP-CaM recovery at 1.8-2.8 s after photobleaching is significantly lower in presence of CFP-Ng than that in presence of CFP alone (38.86 ± 3.12% for CFP-Ng and 55.74 ± 3.56% for CFP, p < 0.05). The effect of Ng on CaM recovery rate indicates an important role for Ng in regulating CaM diffusion, and therefore availability, in dendritic spines. Interestingly, however, the immobile fraction of YFP-CaM (measured when recovery is plateaued) was not affected by the presence of Ng (20.37 ± 1.78% with CFP co-expression and 19.74 ± 2.94% with CFP-Ng co-expression, p = 0.85, Fig. 2E). The finding that Ng did not increase the immobile fraction of CaM indicates that the Ng binding to CaM is dynamic and that Ng does not retain or sequester CaM in spines.

### Spine size is not altered by Neurogranin or Calmodulin overexpression

**Figure 3:**
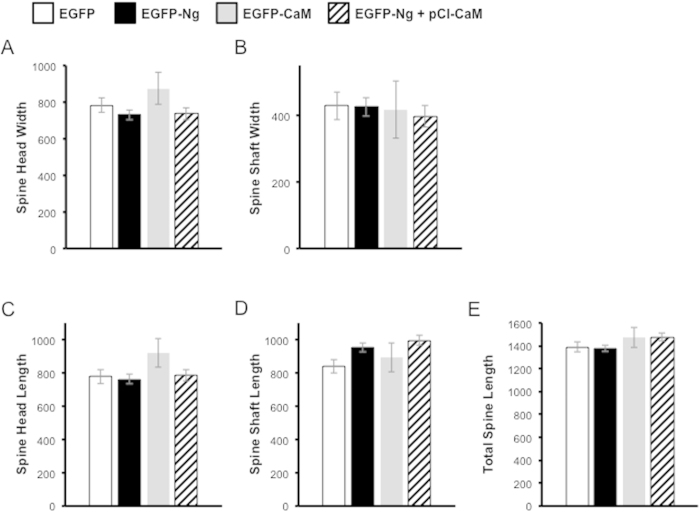
Spine Size is Not Altered by Ng or CaM Overexpression. The bar graphs represent average dendritic spine dimensions measured from neurons expressing EGFP (n = 9), EGFP-Ng (n = 13), EGFP-CaM (n = 17) or EGPF-Ng with pCI-CaM (n = 16). (**A**) The bar graph shows the width of dendritic spine heads in neurons expressing EGFP (784 ± 41 nm), EGFP-Ng (731 ± 28 nm), EGFP-CaM (875 ± 87 nm) and EGFP-Ng with pCI-CaM (737 ± 33 nm) (p = 0.6). (**B**) The bar graph shows the width of dendritic spine shafts in neurons expressing EGFP (429 ± 15 nm), EGFP-Ng (425 ± 13 nm), EGFP-CaM (418 ± 14 nm) and EGFP-Ng with pCI-CaM (378 ± 13 nm) (p = 0.3). (**C**) The bar graph shows the length of dendritic spine heads in neurons expressing EGFP (777 ± 38 nm), EGFP-Ng (763 ± 29 nm), EGFP-CaM (921 ± 48 nm) and EGFP-Ng with pCI-CaM (785 ± 32 nm) (p = 0.1). (**D**) The bar graph shows the length of dendritic spine shafts in neurons expressing EGFP (840 ± 72 nm), EGFP-Ng (952 ± 150 nm), EGFP-CaM (892 ± 77 nm) and EGFP-Ng with pCI-CaM (996 ± 106 m) (p = 0.7). (**E**) The bar graph shows the total length of dendritic spines in neurons expressing EGFP (1389 ± 89 nm), EGFP-Ng (1379 ± 116 nm), EGFP-CaM (1472 ± 89 nm) and EGFP-Ng with pCI-CaM (1475 ± 97 nm) (p = 0.5).

As a control, we wished to monitor if Ng or CaM overexpression alter spine size. To determine whether dendritic spine size and dimensions are altered by protein overexpression, spines were measured in cells expressing EGFP-Ng and/or EGFP-CaM. These conditions were compared to control cells expressing EGFP alone. Figure 3 shows that the expression of Ng and/or CaM does not significantly change any of the parameters measured related to spine structure (i.e. spine head width, shaft width, spine head length, spine shaft length, or the total length of dendritic spines. It is also worth noting that the overexpression of Ng and/or CaM does not change passive neuronal properties^26^.

### Neurogranin concentrates calmodulin at the synapse

**Figure 4:**
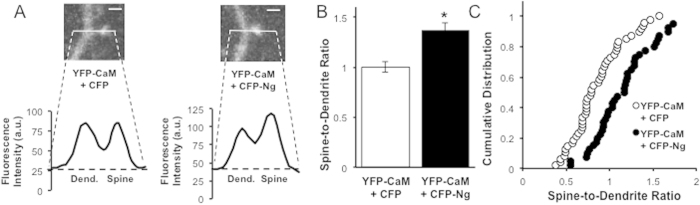
Ng Concentrates CaM in Dendritic Spines. (**A**) Representative confocal image of a spine and the adjacent dendritic shaft from a neuron transfected with YFP-CaM and CFP (left) or with YFP-CaM and CFP-Ng (right), as indicated. Scale bar: 1 μm. Lower panels: representative line plot profiles of YFP fluorescence intensities across dendrite–spine pairs. (**B**) Spine-to-dendrite ratios are calculated from the corresponding peaks of fluorescence intensity at spines and adjacent dendritic shafts. Fluorescence intensity of YFP-CaM in spines was measured and normalized to fluorescence in adjacent dendrite area in the presence of either CFP or CFP-Ng (1.00 ± 0.05 and 1.37 ± 0.07, respectively, p < 0.0001). (**C**) Cumulative distribution of YFP-CaM fluorescence spine-to-dendrite ratio. A right shift of the distribution indicates an overall increase of YFP-CaM spine-to-dendrite ratio in the presence of CFP-Ng.

We also wished to test whether increasing Ng concentration would increase CaM levels within dendritic spines. To directly test whether Ng increases CaM concentration in dendritic spines, we have co-expressed YFP-CaM with CFP-Ng or CFP alone and measured the spine-to-dendrite ratio of YFP fluorescence. Figure 4B shows that the spine-to-dendrite ratio of CaM is significantly increased (36.99 ± 0.07%, p < 0.0001) by Ng co-expression. Figure 4C shows that the cumulative distribution of YFP-CaM spine-to-dendrite ratios is shifted to the right by CFP-Ng co-expression. These data demonstrate that increasing Ng increases levels of CaM at dendritic spines.

### Role of phosphorylation in calmodulin dynamics

**Figure 5:**
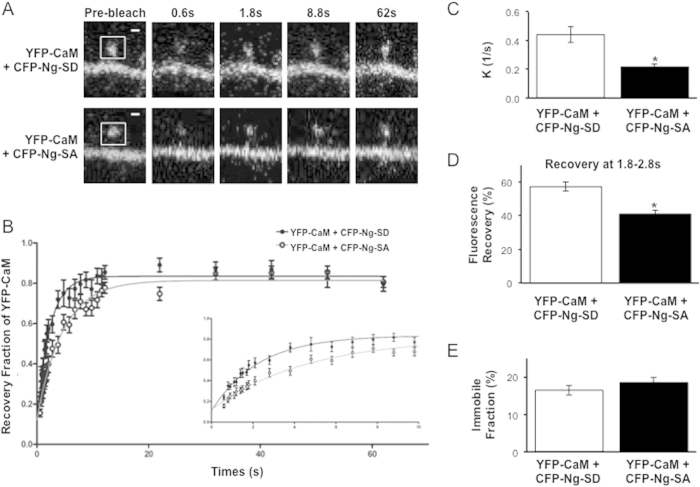
Ng Phosphorylation and CaM dynamics. (**A**) Examples of dendritic spines from neurons co-expressing YFP-CaM with either CFP-Ng-SD (top) or CFP-Ng-SA (bottom) and undergoing FRAP. Representative confocal images are shown before photobleaching (“pre-bleach”), right after photobleaching (“0.6 s”) and at different times during fluorescence recovery, as indicated. Bleached regions are indicated with squares in the “pre-bleach” panels. (**B**) Recovery curves of YFP-CaM in the presence of CFP-Ng-SD or CFP-Ng-SA were fit with a one-phase exponential decay equation (n = 14 CFP-Ng-SD, n = 20 CFP-Ng-SA). Inset shows the initial 10 seconds of fluorescence recovery. (**C**) The bar graph represents the recovery constant (K) of YFP-CaM in presence of CFP-Ng-SD or CFP-Ng-SA (0.44 ± 0.05/s and 0.22 ± 0.02/s, respectively, p < 0.0001. (**D**) The bar graph shows the percent recovery of YFP-CaM fluorescence at 1.8–2.8 s after photobleaching in the presence of CFP-Ng-SD (57.25 ± 2.54%) or CFP-Ng-SA (41.10 ± 2.40%, p < 0.0001). (**E**) The bar graph shows immobile fraction of YFP-CaM fluorescence at recovery plateau in the presence of CFP-Ng-SD (16.5 ± 1.33%) or CFP-Ng-SA (18.57 ± 1.47%, p = 0.3203).

A major postsynaptic modification of Ng is phosphorylation at serine 36 (S36) by Protein Kinase C (PKC). This is important for synaptic plasticity since the phosphorylation state of Ng has been shown to fine-tune the level of potentiation in response to Ca^2+^ influx^39^. Moreover, Ng phosphorylation provides a point of cross-talk between two independent pathways involved in LTP, mGluR- and N-methyl-D-aspartate receptor (NMDAR) -dependent signaling. It has been proposed and demonstrated biochemically that Ng binds to CaM in its non-phosphorylated form, and that phosphorylation causes CaM dissociation^26^,^40^. For example, using pull-down assays, we have shown that a serine to aspartic acid (S36D) mutation in Ng, which provides a positive charge that mimics the phosphorylation, prevents NgS36D from binding to CaM. On the other hand, a serine to alanine (S36A) mutation (i.e. NgS36A) binds CaM in a similar manner to wild type Ng^26^. Interestingly, there is no data to indicate whether there is a differential binding between the phosphorylated or the non-phosphorylated neurogranin to CaM in living neurons. To directly test whether Ng phosphorylation influences CaM diffusion at dendritic spines, we have performed the FRAP assay on YFP-CaM while co-expressing mutations of the Ng phosphorylation site, serine 36. Unlike CFP-Ng-SD, CFP-Ng-SA was able to significantly slow the recovery of YFP-CaM (the rate constant (K) of YFP-CaM is 0.22 ± 0.02/s in the presence of CFP-Ng-SA vs. 0.44 ± 0.05/s in the presence of CFP-Ng-SD, p < 0.0001 (Fig. 5C). Figure 5D shows that CaM recovery at 1.8–2.8 s after photobleaching is significantly lower in presence of Ng-SA than that in presence of Ng-SD (41.10 ± 2.40% for CFP-Ng-SA and 57.25 ± 2.54% for CFP-Ng-SD, p < 0.0001). These data show that, similar to wild-type Ng, Ng-S36A slows the recovery of CaM at dendritic spines. This strongly suggests that Ng-S36A, binds and regulates CaM in dendritic spines in the same manner as wild-type Ng. On the other hand, Ng-S36D does not affect the recovery rate of CaM (0.51 ± 0.08/s for YFP-CaM + CFP, 0.44 ± 0.05/s for YFP-CaM + CFP-Ng-SD, p = 0.5). Figure 5E shows that, similar to Ng, neither mutation influenced the immobile fraction of CaM.

## Discussion

Calmodulin signaling is essential for synaptic plasticity. It mediates Ca^2+^ signaling through its ability to modulate the functions of a variety of proteins in a Ca^2+^-dependent manner. Cellular CaM concentration has been reported to be approximately half the levels of its target proteins, suggesting that CaM availability is limiting^11^,^12^. Thus, the regulation of CaM availability is critically important for controlling CaM signaling. Despite its importance in synaptic function, CaM dynamics and its regulation in dendritic spines have been largely unexplored. We have found that only a small fraction of CaM in dendritic spines is immobile and its diffusion and concentration in spines is modulated by Ng.

An essential mechanism of CaM regulation involves a family of proteins, calpacitins, which bind apo-CaM. The main function of calpacitins, such as Ng, is thought to be their ability to modulate CaM availability. Through their regulated binding to CaM, calpacitins influence synaptic function and plasticity^20^,^22^,^26^,^27^,^41^,^42^,^43^,^44^. Two opposing models exist for CaM regulation by calpacitins. One model is that calpacitins bind to apo-CaM, effectively sequestering CaM to prevent its activation of downstream targets such as the Ca^2+^/CaM-dependent enzymes CaMKII and calcineurin^18^,^19^. An alternative model is that calpacitins can act to target CaM, spatially regulating it in order to facilitate activation of various signaling pathways^20^,^21^,^22^. In the current study, we have tested the effect of an abundant neuronal calpacitin with known effects on synaptic function and plasticity, Ng, on CaM diffusion. Our current finding that Ng does not change the immobile fraction of CaM provides evidence against the model of CaM sequestration. If Ng were acting to sequester CaM, it would be expected to restrain Ng-bound CaM in spines and increase its immobile fraction. Furthermore, our finding that Ng co-expression slows the diffusion of CaM supports the targeting model. By slowing the diffusion of CaM in spines, increased Ng would increase the availability of CaM, which is further supported by our finding that increasing Ng levels increases CaM concentration at dendritic spines. Our current findings agree with previous data regarding the role of Ng in synaptic function, indicating that increased Ng targets CaM within the synapse, enhancing the sensitivity of the synapse to Ca^2+^ influx^26^,^27^. Along with previous data showing that Ng targets a portion of synaptic CaM near the plasma membrane, our current findings strongly support a model in which Ng targets CaM to facilitate Ca^2+^/CaM signaling^27^.

CaM is thought to be rate limiting for CaM-dependent signaling. Therefore, regulation of CaM diffusion within dendritic spines by Ng is likely to have a significant influence on synaptic signaling, including the molecular mechanisms that mediate synaptic plasticity. In support of this view, Ng overexpression enhances synaptic strength, increases CaMKII activation and lowers the threshold for induction of long-term potentiation through the NMDAR-CaMKII pathway^26^,^27^. This indicates that the regulation of CaM dynamics by Ng has physiologically relevant effects.

An important post-translation modification of Ng is phosphorylation by PKC at a serine within the CaM-binding motif. The relevance of this phosphorylation has been explored using mutations that either prevent or mimic phosphorylation at this site. Biochemical analysis of these mutations shows that phosphorylation interferes with CaM binding^26^. The current study is the first to validate this assumption in living neurons. Due to its inability to bind CaM, expression of the phospho-mimic mutation, Ng-SD, was unable to slow CaM recovery relative to the non-phosphorylatable mutation, Ng-SA. Both the recovery rate and plateau of CaM recovery in the presence of Ng-SA was similar to that in the presence of wild type Ng, while recovery in the presence of Ng-SD resembled that of CaM in the absence of Ng. In agreement with previous data, these data support an important role of Ng phosphorylation in regulating CaM availability. Previous studies of Ng and its phosphorylation support a model in which Ng regulates CaM during plasticity induction in a two-step process. First, the increase in Ca^2+^ results in the dissociation of CaM from Ng. Second, the phosphorylation of Ng by PKC prevents CaM and Ng from quickly re-binding, allowing CaM more time to activate its targets^39^.

A surprising finding in this study is that CaM recovery within dendritic spines is fast, indicating the high level of mobility of CaM within dendritic spines. It is worth noting, however, that the absolute recovery time values measured may be an underestimation of the actual recovery time. This is due to the relatively long bleaching time needed (6 s) to achieve consistent photobleaching. This time needed for photobleaching is consistent with other studies using organotypic hippocampal cultures^45^,^46^.

Our finding that a small fraction of CaM is immobile in dendritic spines is in contrast to previous findings in non-neuronal systems. For example, a study using HEK293 cells found that most CaM is bound to target proteins and therefore is slowly diffusing^15^. Also, FRAP experiments of tagged CaM in smooth muscle cells identified an immobile fraction of approximately 37%^14^. While we have found a much smaller immobile fraction, our results cannot directly be compared to those found in smooth muscle and other cell types. It is important to consider the disparity in CaM-binding proteins in non-neuronal cells versus neuronal cells. For example, many calpacitins, such as Ng, are neuron-specific proteins. Furthermore, dendritic spines provide highly-specialized biochemical compartmentalization. Proteins that determine the immobile fraction in smooth muscle and other cells may be absent from neurons or excluded from dendritic spines. Moreover, these different types of cells may have distinct CaM signaling requirements and differential mechanism of CaM regulation. Importantly, finding a small immobile fraction at dendritic spines suggests that CaM interactions in spines are transient and not sufficient to sequester CaM.

In models of CaM signaling, where CaM is thought to be largely immobile, activation of a CaM target is often thought to be at the expense of activation of other targets or pathways. For instance, in endothelial cells, CaM-dependent activation of endothelial nitric oxide synthase was found to be accompanied by a decrease in the activity of a CaM-dependent Ca^2+^ pump^47^. This was found to be due to a reduced amount of CaM available for pump activation. The current findings, where only a small fraction of CaM is found to be immobile at dendritic spines, raise the question of whether a different model may apply to dendritic spines. For example, at dendritic spines, two CaM-dependent enzymes, namely CaMKII and calcineurin, are important for two seemingly opposing functions of the synapse (i.e. insertion and removal of AMPA receptors). We have previously shown that increasing Ng enhances CaMKII activation in a CaM-dependent manner^26^. It remains to be answered whether this increase in CaMKII activation is at the expense of another enzyme, namely calcineurin.

In conclusion, our results provide insight into the dynamics of CaM in dendritic spines and its regulation by Ng. Using living neurons, we show that only a small fraction of CaM in dendritic spines is immobile. The current finding that Ng slows CaM diffusion without altering the immobile fraction of CaM further supports a model in which Ng targets CaM, rather than sequestering it. This is in agreement with our previous findings that Ng enhances synaptic strength through regulated binding to CaM and shifts a pool of CaM closer to the synaptic membrane.

## Additional Information

**How to cite this article**: Petersen, A. and Gerges, N. Z. Neurogranin regulates CaM dynamics at dendritic spines. *Sci. Rep.* **5**, 11135; doi: 10.1038/srep11135 (2015).
